# Systems Pharmacology-Dissection of the Molecular Mechanisms of Dragon's Blood in Improving Ischemic Stroke Prognosis

**DOI:** 10.1155/2020/4858201

**Published:** 2020-05-17

**Authors:** Meng Jiang, Xing Su, Jianling Liu, Chunli Zheng, Xiaogang Li

**Affiliations:** ^1^Precision Medicine Center, The First Affiliated Hospital of Xi'an Jiaotong University, 277 Yanta West Street, Xi'an 710061, China; ^2^Key Laboratory of Xinjiang Phytomedicine Resource and Utilization, Ministry of Education, Shihezi University, Shihezi 832002, China; ^3^Key Laboratory of Resource Biology and Biotechnology in Western China, Ministry of Education, School of Life Sciences, Northwest University, Xi'an 710069, China

## Abstract

**Materials and Methods:**

(1) Based on system-pharmacology platform, the potential active compounds of DB are screened out according to ADME. (2) The ischemic stroke-related targets are predicted by utilizing these active compounds as probes, mapping the targets to the CTD database to establish a molecular-target-disease network. (3) To analyze the mechanism of DB treatment for the prognosis of ischemic stroke, we used the Metascape and DAVID databases to construct “ischemic stroke pathways”. (4) PC12 cells were used to explore the protective effect of loureirin B on oxygen-glucose deprivation/reperfusion (OGD/R) injury, and BV-2 cells were used to determine the anti-inflammation effect of 4′,7-dihydroxyflavone.

**Results:**

Finally, we obtained 38 active compounds and 58 stroke-related targets. Network and pathway analysis indicate that DB is effective in the treatment of ischemic stroke by enhancing cell survival and inhibiting inflammatory and antiplatelet activation. In *in vitro* experiments, the main component loureirin B promoted the expression of HO-1 and Bcl-2 via positive regulation of PI3K/AKT/CREB and Nrf2 signaling pathways in PC12 cells against OGD/R damage. And the anti-inﬂammatory activity of 4′,7-dihydroxyflavone was related to the inhibition of COX-2, TNF-*α*, and IL-6 in LPS-induced BV-2 cells.

**Conclusions:**

In our study, the results illustrated that DB in improving ischemic stroke prognosis may involve enhancing cell survival and antioxidant, anti-inflammation, and antiplatelet activities.

## 1. Introduction

Ischemic stroke is a complex disease caused by the main artery of the brain occludes [1], which provokes neuronal loss [2], leading to great severity of sequelae, such as loss of cognition and paralysis [3]. These sequelae not only seriously affect the patient's quality of life, but also increase the financial burden on the patient's family [4]. For ischemic stroke prognosis, a variety of therapeutic agents have been discovered in recent decades, such as fondaparinux, rivaroxaban, warfarin, and aspirin [5]. There is no doubt that these drugs have a certain effect in the treatment of ischemic stroke prognosis. But the majority of these drugs has a single therapeutic effect and cannot comprehensively treat the complex sequelae caused by the complexity of pathological ischemic stroke and diversity of pathological damage [6, 7].

Dragon's blood (DB) is a rare and precious traditional medicine used by different cultures. It is obtained from different species of four distinct plant genera: *Croton*, *Dracaena*, *Daemonorops*, and *Pterocarpus* [8, 9]. Modern pharmacological studies have confirmed that DB has a wide pharmacology spectrum, such as antiplatelet aggregation [10], stimulating the formation of hematopoietic progenitor cells and improving hematopoietic capacity [11], promoting epidermal growth [12], anti-inflammatory and antioxidative properties [13, 14], and immune suppression and tumors [15]. In recent years, the medicinal standardized phenolic extract of DB has been discovered into a clinical medicine for ischemic stroke, benefiting from its remarkable therapeutic effect [12]. In June 2013, Longxuetongluo capsule (the major ingredient is the total phenolic cluster of DB) was approved by China Food and Drug Administration (CFDA) as a new drug for the treatment of ischemic stroke [16]. However, DB contains more than 80 compounds [17–22] and every compound has different biological activity *in vivo* [20]. The molecular mechanisms and therapy-related signal pathways of DB treated ischemic stroke were still poorly understood at present.

**Figure 1:**
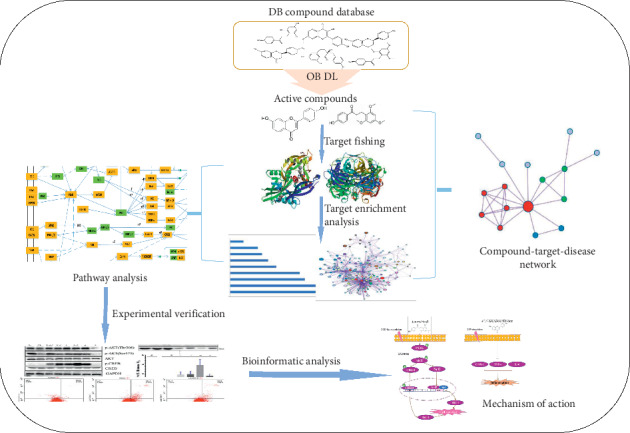
Workflow for systems pharmacology approach.

Recently, systems pharmacology provides an approach to explore the mechanism of treating disease by TCM [22]. It surpasses multilevel complexity and makes a break from molecular and cellular levels to tissue and organism levels [23]. To explore the molecular mechanisms of BD for prognosis treatment after ischemic stroke, a systems pharmacology (as seen in Figure 1) approach was performed. An ADME (i.e., absorption, distribution, metabolism, and excretion) evaluation system was used to screen out the active ingredients of DB with satisfying pharmacokinetics properties. Multiple targets of these active ingredients were captured by the method of similarity ensemble approach (SEA), weighted ensemble similarity (WES), and systematic drug targeting tool [24, 25]. The obtained candidate targets were mapped into TTD and CTD databases to screen out qualified targets corresponding to ischemic stroke. Through the analysis of networks, pathways, and biological processes, we have discovered the potential molecular mechanisms of BD in the treatment of ischemic stroke. To prove the reliability of our method, the hub ingredients of DB were selected to conduct experimental tests at the cellular level.

## 2. Materials and Methods

### 2.1. Database Construction and ADME-Systems Evaluation

A total of 80 chemical ingredients of DB were manually exacted from the TCMSP (http://lsp.nwu.edu.cn/) database [26], which is our own in-house developed database. Considering that glycosides in DB are generally hydrolyzed to dissociation aglycone, which is then absorbed by the intestinal mucosa, we take into account the molecules without glycoligands, which are marked as _qt. All 3D structures of these molecules are saved as mol2 formats.

### 2.2. ADME-Systems Evaluation

In order to obtain the potential bioactive compounds from DB, an ADME incorporated model is used to evaluate the pharmacokinetics and pharmaceutical properties of the obtained compounds, including OB (which predicts oral bioavailability) and DL (which predicts drug-likeness) [27]. The screened active compounds must simultaneously fulfill the two conditions. The constructed prediction model's description is as follows: OB was one of the crucial pharmacokinetics profiles in active compounds screening processes [28]. It also represents a ratio of the orally administered dose that reaches the circulation system under the condition that its activity is unchanged. In this study, a robust system, OBioavail 1.1, was introduced by us to predict OB value for candidate drugs. And the compounds with OB ≥30% were chosen as the candidate molecules [27], according to a previous standard to eliminate poor drug ability compounds in DB. The DL of these ingredients was evaluated by calculating the Tanimoto similarity between herbal compounds and all chemical's average molecular properties in the DrugBank database (http://www.drugbank.ca/) [29]. The PreDL model has been widely used in many studies [30, 31]. In this work, compounds with OB ≥30% and DL ≥0.14 are regarded as bioactive compounds for further analysis.

### 2.3. Target Identification

The compound targeting is based on the *in silico* model that integrates the genomic, pharmacological, and chemical information for compound-target correlations. Based on similarity ensemble approach (SEA) (http://sea.bkslab.org/) [32], weighted ensemble similarity (WES) algorithm [33], and systematic drug targeting tool (SysDT) [34], we synthetically confirm the compound-target interaction. In order to standardize the name of the target, we will target further mapped UniProt (http://www.uniprot.org) [35] database. After standardization, the targets are mapped to Therapeutic Target Database (TTD, http://database.idrb.cqu.edu.cn/TTD) [36] and Comparative Toxicogenomics Database (CTD, http://ctdbase.org/) [37] to obtain their corresponding diseases and screen out potential targets related to ischemic stroke.

### 2.4. Network Construction and Analysis

To clarify the interrelationship between active ingredients and ischemic stroke, the compound-target-disease (C-T-D) network is generated by Cytoscape 3.6.0 [38], a frequently used bioinformatics package for network visualization and data integration. In this section, two plugins, Network Analyzer and CentiScaPe1.2, were used to analyze the quantitative properties of the network. In our graphic network, active compounds, potential targets, ischemic stroke-related diseases, and disease category are represented by nodes, and the edges represented interactions between them. Besides, a vital topological parameter degree was proposed by the plugin Network Analyzer of Cytoscape; the degree was the important parameter for screening targets of the compound; we just take the degree for network analysis as the previous study. The size of nodes represents the degree defined as the number of edges connected to the node.

### 2.5. Functional Enrichment Analysis for Targets

To probe the involved biological processes of the obtained targets, in this work, the targets set were screened out for functional enrichment analysis, staging by Metascape (http://metascape.org). Gene Ontology (GO) enrichment analysis for the biological process (BP) and Kyoto Encyclopedia of Genes and Genomes (KEGG) pathways were enriched. Only terms with *P* value < 0.01 and a number of enriched genes ≥3 were considered as important. Based on their similarities, all the resultant terms were then grouped into clusters. The richest terms in the cluster are presented as representations.

### 2.6. Pathway Constructions and Analysis

At the pathway level, to explore how our mapped targets affect the diseases through responding to specific pathways, an incorporated “*ischemic stroke-related pathway*” was integrated based on the present cognition of ischemic stroke pathology. The obtained human protein targets related to ischemic stroke are gathered and put into the KEGG (http://www.kegg.jp/) [39] database to acquire the information of pathways. The enriched targets-related KEGG pathways with *P*value ≥ 0.05 by Fisher's exact test in the DAVID database (https://david.ncifcrf.gov/) [40] were examined. Finally, based on the information of target pathways derived from DAVID, an incorporated pathway was established to further analyze the relationship between ingredients and disease.

### 2.7. Experimental Validation

#### 2.7.1. Sample Preparation and Cell Cultures

Chemicals 4′,7-dihydroxyflavone and loureirin B were purchased from Shanghai Yuanye Bio-Technology Co., Ltd. (Shanghai, China). The test sample was dissolved in dimethyl sulfoxide (DMSO) at a concentration of 10 mM. The final DMSO (Sigma, USA) concentrations never exceeded 0.1%, which ensured that there was no influence on cell viability.

PC12 cells and BV-2 cells were purchased from China Infrastructure of Cell Line Resources, Chinese Academy of Medical Sciences. PC12 cells were cultured in Dulbecco's Modified Eagle Medium (DMEM, Gibco BRL, USA) supplemented with 10% foetal bovine serum (FBS, Gibco BRL, USA). The BV-2 microglial cells were cultured in DMEM (containing 25 mM HEPES) supplemented with 10% FBS. For inflammation model, BV-2 cells were stimulated with LPS (2 *μ*g/ml). The medium was replaced every 2-3 days, and the cells were passaged at approximately 80–90% confluency. At 80–90% confluence, cells were digested by 0.25% trypsin and were subcultured into 75 cm^2^ flasks.

#### 2.7.2. Cell Viability Assay

The cell viability assay was measured using Cell Counting Kit-8 (CCK-8) (BestBio, Shanghai, China). Briefly, cells were seeded into 96-well flat bottom culture plates at a density of 5 × 10^3^ cells/100 *μ*L per well. After being cultured for 12 h, cells were treated with 100 *μ*l of fresh medium or 100 *μ*l of different concentrations of gradients of 4′,7-dihydroxyflavone and loureirin B dissolved in the medium for an additional 24 h at 37°C in a humidified incubator. After 24 h of incubation, 10 *μ*L of CCK-8 was added to each well. After 3 h of incubation at 37°C and 5% CO_2_, the OD at 450 nm was read on a microplate reader (Molecular Devices, California, USA).

#### 2.7.3. Cell Model Establishment

PC12 cell oxygen-glucose deprivation/reperfusion-induced model: For the purpose of simulating ischemic stroke *in vitro*, 1 × 10^6^ cells were inoculated in the 100 mm culture dish. On the second day of differentiation, we removed the cell culture DMEM medium and washed cells with glucose-free DMEM (Gibco BRL, USA). OGD/R groups were incubated in 5% CO_2_, 95% N_2_, and 0.2% O_2_ at 37°C for 4 h with glucose-free and serum-free DMEM. After 4 h oxygen-glucose deprivation, the cells were treated with the final concentrations of 0.5, 1, 5, 10, and 20 *μ*M of loureirin B and were maintained for 1 h or treated with 10 *μ*M loureirin B for 0.25 h to 4 h. The loureirin B was diluted in serum-free and high-glucose DMEM. BV-2 cell inflammation model: BV-2 cells (1 × 10^6^/well) were cultured in 100 mm culture dish for 24 h and treated with the various concentrations of 4′,7-dihydroxyflavone for 2 h. They were then incubated with 1 *μ*g/mL lipopolysaccharide (LPS from Sigma, USA) for 18 h. The cells were collected at the end of the culture for western blot assays and supernatant for ELISA assays.

#### 2.7.4. Western Blot Assays

Western blotting was performed as described previously [41]. The specific first antibodies GAPDH (1 : 1000), p-AKT (1 : 1000), p-CREB (1 : 1000), CREB (1 : 1000), AKT (1 : 1000), COX-2 (1 : 1000), TNF-*α* (1 : 1000), HO-1 (1 : 1000), Nrf2 (1 : 1000), iNOS (1 : 1000), and Histone H_3_ (1 : 5000) were purchased at CST (Cell Signaling Technology, INC). After three times of wash with TBST (pH = 7.2), for 10 minutes each, membranes were incubated with horseradish peroxidase-conjugated secondary mouse anti-rabbit IgG (1 : 15,000 dilutions) for 1 hour at room temperature. The membranes were washed three times, 10 minutes/time; the immunoreactive bands were then detected using ECL Detection Kit (Bio-Rad Laboratories, Richmond, California, USA), and labelling was visualized by Image Lab software (Bio-Rad).

#### 2.7.5. Flow Cytometric Analysis

Cells were plated in six-well dishes at a density of 3 × 10^6^ per well. After 24 h culture, medium was removed and cells were collected by trypsinization. Cells were washed, resuspended in cold binding buffer, and treated according to the protocol of FITC Annexin V Apoptosis Detection Kit I (BD556547 from the USA). After 15 min incubation at room temperature in dark, cells were detected by flow cytometry.

#### 2.7.6. Measurement of IL-6 and TNF-*α* Production

The ELISA Kit from R&D Systems (Minneapolis, MN, USA) was used for the measurement of TNF-*α* (VAL609) and IL-6 (VAL604), according to the protocols of the manufacturer. Briefly, after collecting and centrifuging the supernatant of the cell culture, 50 *μ*L of samples was added to each well for ELISA. The samples' concentration was calculated according to the standards provided in the kit.

### 2.8. Statistical Analysis

The statistical results were analyzed by one-way ANOVA, Student's *t*-test, and post hoc analysis (GraphPad Prism version 7). Western blot and flow cytometric analysis were repeated three times in independent experiments with the same result. In the figures, the standard symbols were used: ^*∗*^*P* <  0.05,^*∗∗*^*P* < 0.01,^*∗∗∗*^*P* < 0.001,  and ^*∗∗∗∗*^*P* < 0.0001.

## 3. Results

### 3.1. Compound-Target-Diseases (C-T-D) Network Construction and Analysis

Potential active components of DB have been screened out using the ADME system. The results of screening showed that 38 compounds (as shown in Supplementary Table 1) reached the standard of OB ≥30%, DL ≥0.14. It includes 12 flavonoids, 9 chromones, 6 chalcones, 4 phenols, 8 terpenoids, and phytosterols. Some of these 38 active compounds have been demonstrated with significant pharmacological activity. For instance, pterostilbene (MOL060, OB = 77.8%, DL = 0.14) has been reported with antioxidation, antiapoptosis, and neuroprotective effects. It can also inhibit the Nox2-related oxidative stress and NLRP3 inflammasome to attenuate early brain injury in C57BL/6 J mice [42]. Loureirin A (MOL004, OB = 64.5%, DL = 0.18) has the effect of antiplatelet aggregation via inhibiting PI3K/AKT signaling pathway in platelets [43]. These candidate compounds might be the key elements for ischemic stroke.

**Figure 2:**
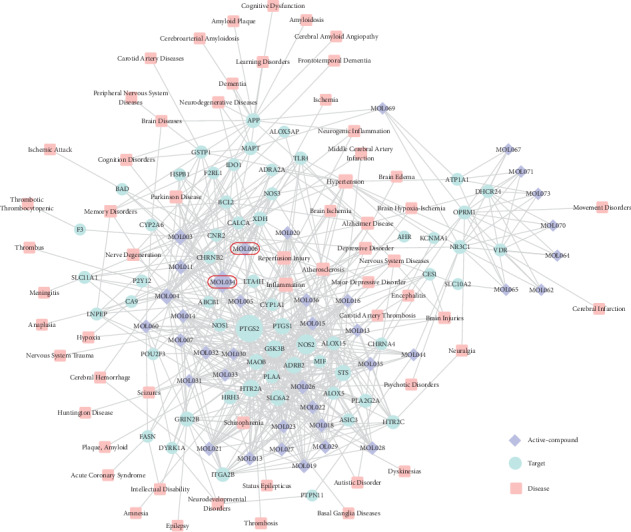
C-T-D network. A compound node and a protein node are linked if the protein is targeted by the corresponding compound; a protein node and disease node are linked if disease is regulated by the corresponding protein target. Node size is proportional to its degree. The frame represents our experimental selection molecule.

We obtained 171 candidate targets for 38 active compounds using WES and SysDT methods. The results show that most compounds can map into more than one target. Through the CTD and TTD database analysis, 58 potential targets and 61 ischemic stroke-related diseases were acquired after deleting repetition. After targets and disease enrichment analysis, we constructed the compound-target-disease network (C-T-D network) to visualize their relationship (as shown in Figure 2). The C-T-D network consists of 38 active compounds, 58 targets, and 61 diseases.

### 3.2. Functional Characterization of Targets

**Figure 3:**
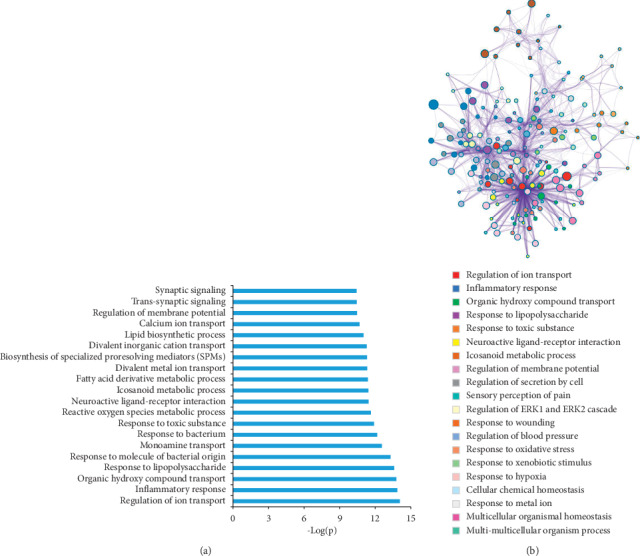
Metascape pathway and process enrichment analysis. (a) The top 20 clusters with their representative enriched terms (one per cluster), for each given gene list, pathway, and process enrichment analysis are carried out with the following ontology sources: KEGG pathway, GO biological processes, Reactome gene sets, canonical pathways, and CORUM. All genes in the genome are used as the enrichment background. Terms with a *Pvalue < 0.01*, a minimum count of 3, and an enrichment factor >1.5. (b) To further capture the relationships between the terms, a subset of enriched terms are selected and rendered as a network plot, where terms with a similarity > 0.3 are connected by edges. We select the terms with the best *P* values from each of the 20 clusters, with the constraint that there are no more than 15 terms per cluster and no more than 250 terms in total. The network is visualized using Cytoscape, where each node represents an enriched term and is colored by cluster ID.

In order to detect the correlation between targets and ischemic stroke, we performed enrichment analysis by Metascape (http://metascape.org) [44] (Figure 3).  Figure 3(a) shows the top 20 clusters with their representative enriched terms (one per cluster). To further capture the relationships between the terms, a subset of enriched terms has been selected and rendered as a network pilot, where terms with a similarity >0.3 are connected by edges. We select terms with the best *P* values from each of the 20 clusters (Figure 3(b)). The targets mainly involve biological processes such as inflammatory response, response to lipopolysaccharide, and neuroactive ligand-receptor interaction.

### 3.3. Ischemic Stroke Pathway Construction and Analysis

**Figure 4:**
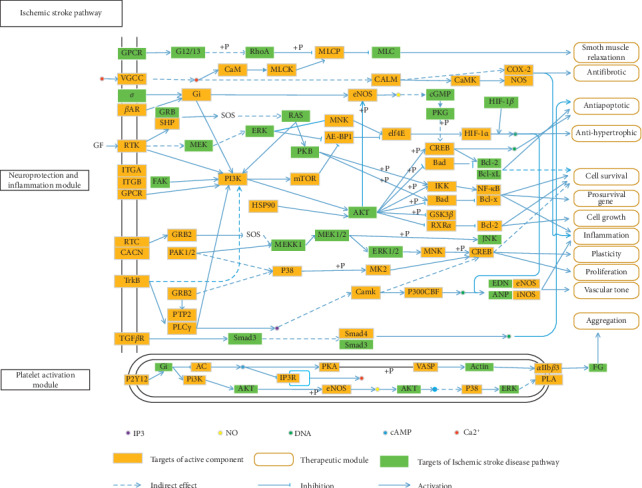
The representative ischemic stroke pathway and therapeutic modules of DB.

To explain the mechanisms of DB treatment of ischemic stroke in a pathway level, 56 pathways (as shown in Supplementary Table 2) were obtained by mapping the active targets to the KEGG database. An incorporated “ischemic stroke pathway” was constructed by integrating the pivotal pathways obtained from pathway enrichment. The “ischemic stroke pathway” contains 58 protein targets of the active compounds (Figure 4). This pathway exhibits two modules: neuroprotection and inflammation, platelet activation. Here, we mainly concentrate on these two modules to reveal the underlying therapeutic effects of the DB.

#### 3.3.1. Neuroprotection and Inflammation Module

Our results show that the active compounds from DB closely relate to PI3K, Bcl-2, COX-2, p38, CREB, GSK3*β*, and TrkB, most of which were associated with oxidative stress and brain inflammation cause of ischemic stroke. COX-2 (cyclooxygenase-2) protein factor was important to regulate brain injury and inflammation after ischemic stroke [45]. Increasing phospho-AKT (protein kinase B) after ischemic stroke can upregulate the phosphorylation level of cAMP response element binding protein (CREB) [46], which has been demonstrated as a key transcription factor in neuroprotection to decrease brain damage [47]. P38MAPK (p38 mitogen-activated protein kinase) plays a key role in the cerebral ischemic stroke process [48]; continuously activated p38MAPK can promote inflammation, causing damage aggravation after ischemic stroke [49]. Antiapoptosis protein Bcl-2 (B cell lymphoma 2) is a member of Bcl-2 protein family. Highly expressed Bcl-2 can protect against cell apoptosis [50]. A key player, glycogen synthase kinase-3 beta (GSK3*β*), is a regulation factor in neuronal survival. Inhibition level of GSK3*β* in different survival factors is a common event in neuroprotection [51]. Tyrosine kinase B (TrkB) is a key target for neural survival and growth activated by brain-derived neurotrophic factor (BDNF) [52]. The above analysis results indicate that DB may protect neural cells from ischemic damage and anti-inflammation in the brain to treat ischemic stroke.

#### 3.3.2. Platelet Activation Module

Our enrichment result indicates that P2Y12 receptor was the target of loureirin A (MOL004, degree = 24). Inhibition of the P2Y12 receptor can be antiplatelet and antithrombotic; one of the P2Y12 receptor functions is regulation of Ca^2+^ release, which can promote blood coagulation by action of factor Xa [53]. In platelets, PI3K/AKT signaling pathway activity was essential for their aggregation. A study demonstrated that loureirin A showed antiplatelet activity by inhibiting the phosphorylation of AKT (Ser473) [43]. The analysis results indicate that DB may have treated ischemic stroke through inhibition of the P2Y12 and *α*IIb*β*3 receptors, showing antithrombotic activity.

### 3.4. Experimental Validation

**Figure 5:**
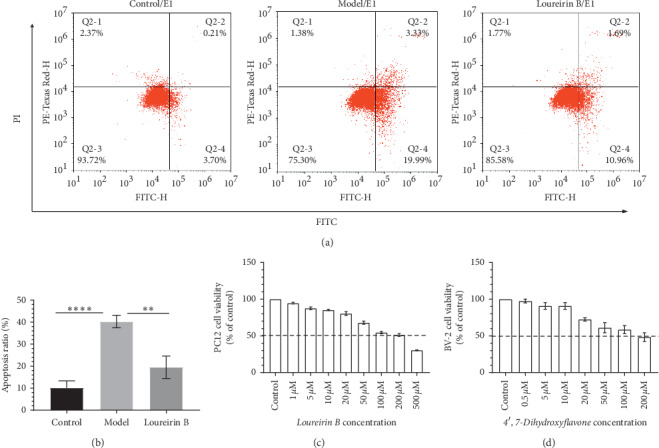
(a), (b) Flow cytometry analysis results: After 4 h OGD, loureirin B group cells are treated with 10 *μ*M loureirin B added to reperfusion culture for 2 h. Cells in model group are only cultured in normal medium for 2 h; cells in control group are not treated. (c) Toxicity of loureirin B to PC12 cells and (d) toxicity of 4′,7-dihydroxyflavone to BV-2 cells. Data in Figures 5(a), 5(b), and 5(c) are presented as the mean ± SD (*n* = 3). ^*∗*^*P* <  0.05,^*∗∗*^*P* < 0.01,^*∗∗∗*^*P* < 0.001,  and ^*∗∗∗∗*^*P* < 0.0001.

To verify the reliability of our analysis, we conducted experimental verification. Significantly, 4′,7-dihydroxyflavone (MOL034, degree = 25) was a hub node compared to other compounds in C-T-D network, and the analysis result shows that the target of 4′,7-dihydroxyflavone can be involved in the inflammatory response. Loureirin B (LB) (MOL006, degree = 19) is one of the most important components of dragon's blood (*Dracaena cochinchinensis* plant) resin [54]. LB was the marker compound of DB, and the targets of loureirin B relate to cell survival and antiapoptosis. Importantly, the Annexin V/PI staining assay indicated that exposure to OGD/R increased PC12 cells apoptosis compared to control group. Conversely, loureirin B treatment effectively decreased the percentage of apoptosis cells in PC12 cells treated with OGD/R (Figures 5(a) and 5(b)). Based on the above reasons, we used an *in vitro* glucose oxygen deprivation/reperfusion injury model to study the molecular mechanism of loureirin B, using BV-2 neuroglial bacterial capsular lipopolysaccharide (LPS, from Sigma USA) to stimulate inflammation models to discuss the molecular mechanism of 4′,7-dihydroxyflavone. The CCK-8 is shown in Figures 5(c) and 5(d); loureirin B and 4′,7-dihydroxyflavone do not cause significant cytotoxicity at the concentrations used (0 to 20 *μ*M for loureirin B and 0 to 20 *μ*M for 4′,7-dihydroxyflavone).

#### 3.4.1. Loureirin B Activated the PI3K/AKT/CREB and Nrf2 Signaling Pathway to Attenuate OGD/R Damage in PC12 cells

**Figure 6:**
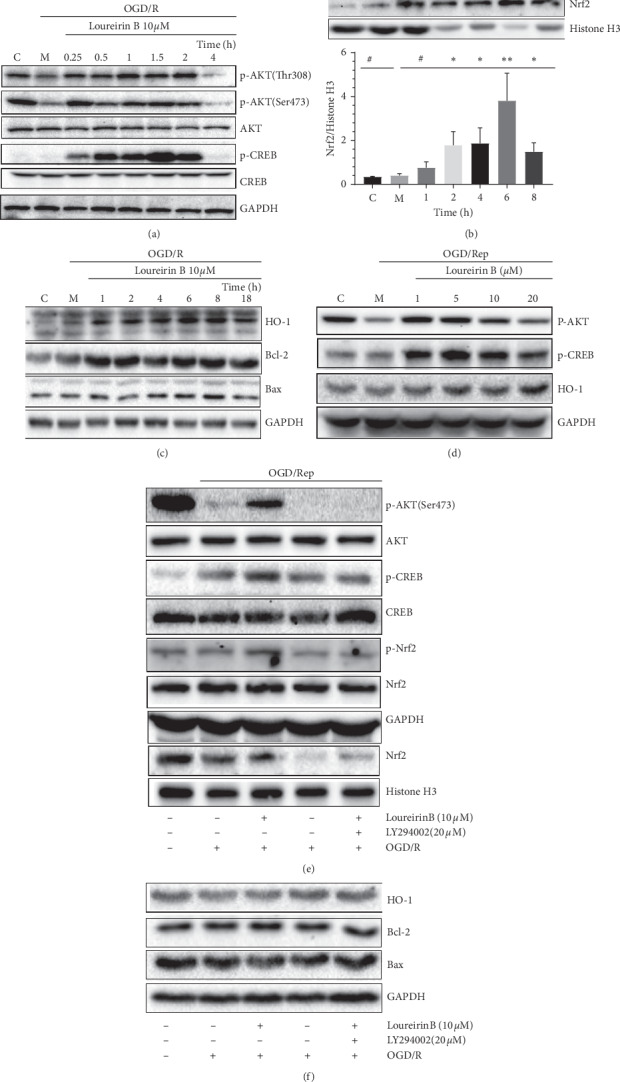
The neuroprotective effects of loureirin B were regulated by AKT, CREB, and Nrf2. (a) PC12 cells were exposed to loureirin B for 0 to 4 h after 4 h OGD, and then cell extracts were prepared for western blotting. (b) PC12 cells were treated with 10 *μ*M loureirin B for 1 to 8 h after 4 h OGD, then the nucleoprotein was extracted to test the Nrf2. (c) PC12 cells were treated with 10 *μ*M loureirin B reperfusion culture for 1 to 18 h after 4 h OGD. Then, the total protein was extracted with western blotting (d) after 4 h OGD; PC12 cells were treated with the different doses of loureirin B for 1.5 h, and then cell extracts were equipped for western blotting. (e) The activation of the pathway by loureirin B was inhibited by LY294002: after 4 h OGD, PC12 cells were treated with loureirin B (10 *μ*M) in the absence and presence of 20 *μ*M LY294002 as indicated for 1.5 h. (f) Inhibition of PI3K in loureirin B-induced HO-1 expression and Nrf2 nuclear translocation. After 4 h OGD, cells were incubated with 10 *μ*M loureirin B in the absence and presence of 20 *μ*M LY294002 for 6 h. Data in Figure 6(b) are presented as the mean ± SD (*n* = 3). ^*∗*^*P* <  0.05,^*∗∗*^*P* < 0.01,^*∗∗∗*^*P* < 0.001,  and ^*∗∗∗∗*^*P* < 0.0001.

To determine the mechanism of loureirin B against OGD/R cells, we study the effect of loureirin B treatment time on the phosphorylation level of the AKT/CREB pathway. After 4 h of OGD, the final concentration of 10 *μ*M loureirin B was added to reperfusion culture for 0 to 4 h, and cytosolic proteins and nuclear proteins were extracted from the cells according to the kit requirements for western blotting analysis. The results show that the phosphorylation levels of AKT (Ser473) increased in a time-dependent manner, and the phosphorylation level was the highest at the 1.5 h time point. Phosphorylation of CREB increase in level also shows a time dependence trend compared to the model group and the control group (Figure 6(a)). Furthermore, we observed in the nuclear protein WB assay that nuclear factor-E2-related factor-2 (Nrf2) accumulates in the nucleus in a time-dependent manner (Figure 6(b)). It has been demonstrated that nuclear translocation of Nrf2 can upregulate HO-1 expression, which is one of phase II detoxifying enzymes and plays important roles in antioxidation protection in ischemia/reperfusion. Our result proves that the expression level of HO-1 could be upregulated by loureirin B in a time-depend manner (Figure 6(c)).

To further confirm that the phosphorylation levels of AKT and CREB are affected by the concentration of the loureirin B. We used 1 to 20 *μ*M loureirin B to treat PC12 cells by 1.5 h. The results of the western blotting assay indicate that the phosphorylation levels of AKT and CREB were closely related to the concentration of administration, and the phosphorylation level was most significant at a concentration of 5 *μ*M. The expression level of HO-1 also correlated with the concentration of loureirin B and significantly increased in 5 to 20 *μ*M concentration and the 6 h time point (Figure 6(d)). These results clearly demonstrate that the PI3K/AKT/CREB and Nrf2 signaling pathway can be activated by loureirin B in time- and concentration-dependent manner.

To demonstrate whether phosphorylation activation of AKT is induced by PI3K, we applied the PI3K specific inhibitor LY294002 [55] to determine AKT phosphorylation change after loureirin B treatment. PC12 cells were aerobically cultured for 1.5 h after 4 h OGD, and normal sugar medium containing loureirin B (10 *μ*M) and LY294002 (20 *μ*M) was added. As shown in Figure 6(e), the phosphorylation level of AKT (Ser473) was significantly inhibited compared to the loureirin B group.

The phosphorylation levels of CREB and Nrf2 were also affected by LY294002, which is significantly lower than that of the loureirin B group, and the nuclear accumulation of Nrf2 also showed the same phenomenon. The expressions of HO-1 and Bcl-2 were also reduced by LY294002 (Figure 6(f)). Our results show that phosphorylation of AKT is inhibited by LY294002 and can downregulate the phosphorylation of Nrf2 and CREB and decrease the nuclear translocation of Nrf2. The results also indicate that PI3K is involved in the induction of loureirin B and activates AKT.

Evidence indicates that the phenolic extract of DB can protect against ischemic brain damage in rats and remarkably reduced infarct volume [56]. Therefore, it is possible that DB plays a role in neuroprotection against ischemia/reperfusion. In our *in vitro* ischemic stroke model, loureirin B at concentration of 10 *μ*M significantly protected PC12 cells from oxidative damage. We first found that the loureirin B decreased the OGD/R damage by upregulation expression of HO-1 and Bcl-2 via activating PI3K/AKT/CREB and Nrf2 pathway. Activated Nrf2 modulated the ARE-regulated genes expression, encoding antioxidant enzymes, including HO-1 [55]. In accordance with the results of our study, we demonstrated the loureirin B-induced Nrf2 accumulated in the nucleus and HO-1 expression in a time- and concentration-dependent manner. Further western blotting demonstrated the potential effect of loureirin B on the phosphorylation of AKT and CREB. The CREB is also known as an important nuclear transcription factor that triggers expression of neuroprotective proteins, including and Bcl-2 [50]. In our present study, CREB is rapidly phosphorylated by 5 *μ*M loureirin B and we find that phosphorylated CREB is obviously increased in as early as 0.25 h. The expression level of Bcl-2 in the model group is significantly lower than the control, while the treatment of 10 *μ*M loureirin B is higher than the model group. The intracellular ROS production and proapoptotic protein expression can be inhibited by Bcl-2.

In summary, loureirin B activates the PI3K/AKT/CREB and Nrf2 signaling pathway to upregulate the expression of Bcl-2 and HO-1, which attenuate OGD/R damage in PC12 cells. However, the exact mechanism needs further study.

#### 3.4.2. 4′,7-Dihydroxyflavone Reduces Inflammatory Response of Neuroglia by Downregulating COX-2, IL-6, and TNF-*α*

**Figure 7:**
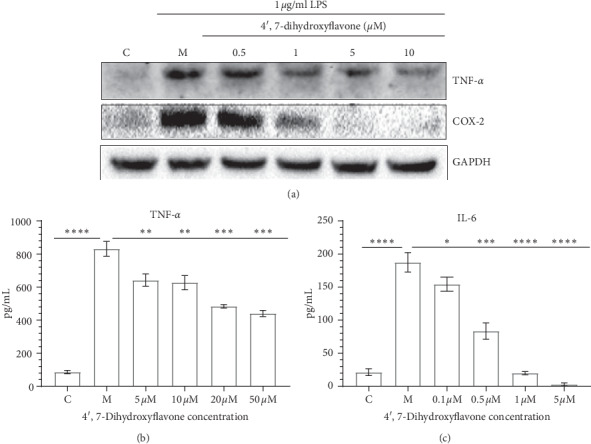
Effect of 4′,7-dihydroxyflavone on LPS-induced production of proinflammatory mediators. (a) Expression of TNF-*α* and COX-2 in BV-2 cells analyzed by western bolt. (b, c) Expression levels of TNF-*α* and IL-6 in BV-2 cells: experience group cells treated with different doses of 4′,7-dihydroxyflavone and analyzed by ELISA. Data are presented as the mean ± SD (*n* = 3). ^*∗*^*P* <  0.05,^*∗∗*^*P* < 0.01,^*∗∗∗*^*P* < 0.001, and ^*∗∗∗∗*^*P* < 0.0001.

The BV-2 cell inflammation model is used to determine the pharmacological activity of 4′,7-dihydroxyflavone. We set up the normal group (C), inflammation model group (BV-2 cells were treated with 1 *μ*g/ml LPS, M), and 4′,7-dihydroxyflavone treatment groups. After 18 hours, cultured BV-2 cells were collected and the total protein was extracted. The result of western blotting analysis showed that the expression of COX-2 was downregulated by 4′,7-dihydroxyflavone. Obviously, COX-2 was most significantly downregulated at 5 *μ*M concentration (Figure 7(a)). Proinflammatory cytokines TNF-*α* and interleukin 6 (IL-6) are considered as markers of activated microglia cells [57, 58]. Therefore, we examined TNF-*α* and IL-6 by ELISA, and our results demonstrate that the 4′,7-dihydroxyflavone can downregulate the levels of TNF-*α* and IL-6 by in a concentration-dependent manner (Figures 7(b) and 7(c)). Consequently, the data suggest that 4′,7-dihydroxyflavone is important to anti-inflammation after ischemic stroke.

Activated microglia after ischemia plays a central role in inflammation brain damage [57]. They can produce proinflammatory (TNF-*α*, IL-6, and COX-2) factors to recruit inflammatory-associated immune cells. In our *in vitro* LPS-induced inflammation model, the results of ELISA assay show that 4′,7-dihydroxyflavone significantly inhibited the production of TNF-*α* and IL-6, especially IL-6. The western blotting assay indicates that the protein expression levels of COX-2, TNF-*α*, and IL-6 are also inhibited by 4′,7-dihydroxyflavone, especially 5 *μ*M concentration point. The mechanism 4′,7-dihydroxyflavone may protect against microglia-mediated neuroinflammation in the brain after ischemia.

## 4. Conclusion

**Figure 8:**
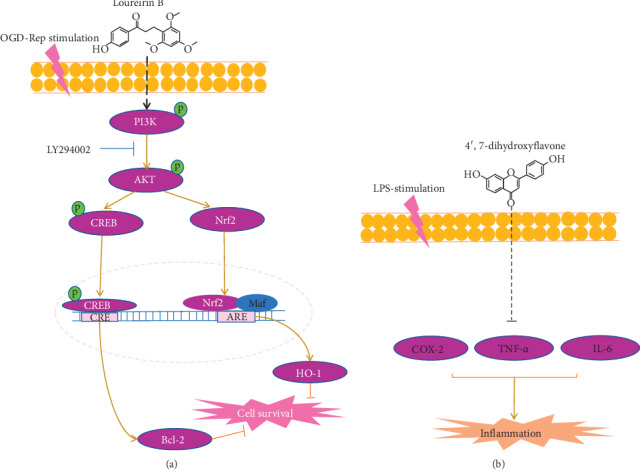
(a) The neuroprotective properties and schematic mechanism of loureirin B against oxygen-glucose deprivation/reperfusion lead to PC12 cell death. (b) The schematic mechanism of 4′,7-dihydroxyflavone anti-inflammation in the BV-2 microglial cells after LPS stimulation.

In the present study, we employed the systems pharmacology method to investigate the deep pharmacological mechanisms of DB. Based on the evaluation method, 38 active compounds were obtained from DB, and 171 potential targets were predicted. The Metascape enrichment analysis shows that DB mainly treats ischemic stroke through its anti-inflammatory and antioxidative stress effects. Ischemic stroke pathway analysis demonstrated that DB can modulate the binding of multiple targets/pathways to many of therapeutic modules, such as anti-inflammatory, antiapoptosis, and antiplatelet activities. Through *In vitro* cell experiments, we verified the mechanisms of active ingredients such as loureirin B and 4′,7-dihydroxyflavone (as shown in Figure 8). Loureirin B activates PI3K/AKT/CREB and Nrf2 signaling pathways, which upregulate the expression of HO-1 and Bcl-2, to protect PC12 cells from OGD/R damage. The 4′,7-dihydroxyflavone, via downregulating the proinﬂammatory factors TNF-*α*, IL-6, and COX-2, inhibits the activated microglia-mediated neuroinflammation response. In conclusion, our results explain the molecular mechanism of DB in treating ischemic stroke and discover the new functions of loureirin B in protecting PC12 cells from OGD/R damage. All of these results provide a reference for future drug development based on DB.

## Data Availability

The data such as active compound, targets, and pathways to support the findings of this study are included within the article. The data such as compound structure, pathway and process enrichment analyses, C-T-D network, Western blot, and flow cytometry, used to support the findings of this study are included within the supplementary information table. The other data used to support the findings of this study are available from the corresponding author upon request because part of the data for this result is included in the mentor's fund project, and it is temporarily not suitable for disclosure.

## Abbreviations

DB: Dragon's blood
TCM: Traditional Chinese medicine
ADME: Absorption, distribution, metabolism, and excretion
OGD/R: Oxygen-glucose deprivation/reperfusion.

## Supplementary Materials

(Supplementary Materials) Table 1: the information of compounds. Table 2: the information of disease-related pathways.
