# Supplementary material for: Systems Pharmacology-Dissection of the Molecular Mechanisms of Dragon's Blood in Improving Ischemic Stroke Prognosis
Source: Evid Based Complement Alternat Med. 2020 May 17;2020:4858201. doi: 10.1155/2020/4858201 (PMC7251463; doi:10.1155/2020/4858201)
Supplement: (Supplementary Materials) — Table 1: the information of compounds. Table 2: the information of disease-related pathways. [file 4858201.f1.docx]

Table. 1 The information of compounds.

| MOL-ID | Compounds | Structure | OB | DL | Degree |
| --- | --- | --- | --- | --- | --- |
| MOL003 | Loureirin D (6-methoxy-2,4,4'-trihydroxydihydrochalcone) |  | 41.25 | 0.19 | 23 |
| MOL004 | Loureirin A (4'-hydroxy-2,4-dimethoxydihydrochalcone) |  | 64.57 | 0.18 | 24 |
| MOL005 | Scutellariae A (4'-hydroxy-2,6-dimethoxydihydrochalcone) |  | 64.86 | 0.18 | 11 |
| MOL006 | Loureirin B (4'-hydroxy-2,4,6-trimethoxydihydrochalcone) |  | 61.52 | 0.23 | 19 |
| MOL007 | 4-hydroxy-2-methoxydihydrochalcone |  | 42.81 | 0.18 | 13 |
| MOL011 | 2'-Methoxy-4,4'-dihydroxychalcone |  | 51.30 | 0.16 | 16 |
| MOL013 | 4',7-dihydroxyflavan |  | 44.85 | 0.16 | 15 |
| MOL014 | 7-Hydroxy-4'-methoxyxanane |  | 30.53 | 0.18 | 13 |
| MOL015 | 4',7-dihydroxy-3'-methoxyflavan |  | 48.14 | 0.21 | 14 |
| MOL016 | 3',7-dihydroxy-4'-methoxyflavanane |  | 47.42 | 0.21 | 13 |
| MOL018 | 4',7-dihydroxy-8-methylflavan |  | 30.21 | 0.18 | 13 |
| MOL019 | 4',7-dihydroxy-8-methyl-3'-methoxyflavan |  | 43.36 | 0.23 | 14 |
| MOL020 | 4',5-dihydroxy-8-methyl-7-methoxyflavan |  | 41.51 | 0.23 | 10 |
| MOL021 | 7-Hydroxy-3-(4-hydroxyphenyl)-chroman |  | 64.73 | 0.18 | 16 |
| MOL022 | 6-Hydroxy-7-methoxy-3-(4-hydroxyphenyl)-chroman |  | 43.16 | 0.24 | 14 |
| MOL023 | 7-Hydroxy-3-(3-hydroxy-4-methoxyphenyl)-chromogen |  | 75.05 | 0.24 | 12 |
| MOL026 | 4',7-dihydroxy-8-methyl high isoflavone |  | 62.16 | 0.24 | 15 |
| MOL027 | 4',5,7-trihydroxy high isoflavone |  | 66.35 | 0.24 | 13 |
| MOL028 | 4',5,7-Trihydroxy-6-methyl high isoflavone |  | 53.82 | 0.27 | 11 |
| MOL029 | 4',7-dihydroxy high isoflavone |  | 57.10 | 0.21 | 13 |
| MOL030 | 3,5,7-trihydroxy-4'-methoxy high isoflavone |  | 56.72 | 0.30 | 8 |
| MOL031 | 4',7-Dihydroxy-5-methoxy high isoflavone |  | 50.34 | 0.27 | 11 |
| MOL032 | Acanthopanax B |  | 47.45 | 0.76 | 12 |
| MOL033 | 3,4'-dihydroxy-7-methoxylated flavones |  | 45.86 | 0.24 | 10 |
| MOL034 | 4',7-dihydroxyflavone |  | 37.31 | 0.24 | 25 |
| MOL035 | 4'-Methoxy-3',7-dihydroxyflavone |  | 74.26 | 0.18 | 15 |
| MOL036 | 7-hydroxydihydroflavone |  | 66.42 | 0.16 | 14 |
| MOL043 | 10-hydroxy-11-methoxydrocaenone (10-hydroxy-11-methoxydracaenone) |  | 69.88 | 0.35 | 11 |
| MOL044 | 7,10-Dihydroxy-11-methoxydragonone (7,10-dihydroxy-11-methoxydracaenone) |  | 62.92 | 0.39 | 8 |
| MOL060 | 4'-hydroxy-3,5-dimethoxydiphenylene (pterostilbene) (Pteralwood) |  | 77.88 | 0.14 | 12 |
| MOL062 | 4-ene-3-cholestone (cholest-4-en-3-one) |  | 37.17 | 0.68 | 4 |
| MOL064 | 4-methyl-7-enecholyl alcohol (4α-methylcholest-7-en-3β-ol) |  | 38.12 | 0.72 | 4 |
| MOL065 | 31-demethylcyclopyrrolidyl alcohol (31-norcycloartanol) |  | 38.10 | 0.79 | 5 |
| MOL067 | cyclopentanol (cycloartanol) |  | 38.25 | 0.78 | 4 |
| MOL069 | 24-methylenecyclopentanol (24-methylenecycloartanol) |  | 38.55 | 0.76 | 5 |
| MOL070 | Cholesterol (cholesterol) |  | 37.87 | 0.68 | 4 |
| MOL071 | Campesterol (campesterol) |  | 37.57 | 0.72 | 5 |
| MOL073 | sitosterol |  | 36.90 | 0.75 | 5 |

Table. 2 The information of disease-related pathways

| Category | Term | Pathways | Degree |
| --- | --- | --- | --- |
| KEGG_PATHWAY | hsa04080 | Neuroactive ligand-receptor interaction | 31 |
| KEGG_PATHWAY | hsa04020 | Calcium signaling pathway | 21 |
| KEGG_PATHWAY | hsa04970 | Salivary secretion | 13 |
| KEGG_PATHWAY | hsa04024 | cAMP signaling pathway | 17 |
| KEGG_PATHWAY | hsa04022 | cGMP-PKG signaling pathway | 15 |
| KEGG_PATHWAY | hsa04913 | Ovarian steroidogenesis | 9 |
| KEGG_PATHWAY | hsa04540 | Gap junction | 11 |
| KEGG_PATHWAY | hsa04726 | Serotonergic synapse | 12 |
| KEGG_PATHWAY | hsa04261 | Adrenergic signaling in cardiomyocytes | 13 |
| KEGG_PATHWAY | hsa04750 | Inflammatory mediator regulation of TRP channels | 11 |
| KEGG_PATHWAY | hsa04915 | Estrogen signaling pathway | 11 |
| KEGG_PATHWAY | hsa00330 | Arginine and proline metabolism | 8 |
| KEGG_PATHWAY | hsa04914 | Progesterone-mediated oocyte maturation | 10 |
| KEGG_PATHWAY | hsa05200 | Pathways in cancer | 22 |
| KEGG_PATHWAY | hsa04976 | Bile secretion | 9 |
| KEGG_PATHWAY | hsa04725 | Cholinergic synapse | 11 |
| KEGG_PATHWAY | hsa04923 | Regulation of lipolysis in adipocytes | 8 |
| KEGG_PATHWAY | hsa00140 | Steroid hormone biosynthesis | 8 |
| KEGG_PATHWAY | hsa04924 | Renin secretion | 8 |
| KEGG_PATHWAY | hsa05031 | Amphetamine addiction | 8 |
| KEGG_PATHWAY | hsa00100 | Steroid biosynthesis | 5 |
| KEGG_PATHWAY | hsa04919 | Thyroid hormone signaling pathway | 10 |
| KEGG_PATHWAY | hsa04972 | Pancreatic secretion | 9 |
| KEGG_PATHWAY | hsa04270 | Vascular smooth muscle contraction | 10 |
| KEGG_PATHWAY | hsa04728 | Dopaminergic synapse | 10 |
| KEGG_PATHWAY | hsa00590 | Arachidonic acid metabolism | 7 |
| KEGG_PATHWAY | hsa05215 | Prostate cancer | 8 |
| KEGG_PATHWAY | hsa04720 | Long-term potentiation | 7 |
| KEGG_PATHWAY | hsa04910 | Insulin signaling pathway | 10 |
| KEGG_PATHWAY | hsa05030 | Cocaine addiction | 6 |
| KEGG_PATHWAY | hsa04971 | Gastric acid secretion | 7 |
| KEGG_PATHWAY | hsa01100 | Metabolic pathways | 40 |
| KEGG_PATHWAY | hsa04611 | Platelet activation | 9 |
| KEGG_PATHWAY | hsa04931 | Insulin resistance | 8 |
| KEGG_PATHWAY | hsa04114 | Oocyte meiosis | 8 |
| KEGG_PATHWAY | hsa05032 | Morphine addiction | 7 |
| KEGG_PATHWAY | hsa04713 | Circadian entrainment | 7 |
| KEGG_PATHWAY | hsa00983 | Drug metabolism - other enzymes | 5 |
| KEGG_PATHWAY | hsa04917 | Prolactin signaling pathway | 6 |
| KEGG_PATHWAY | hsa04723 | Retrograde endocannabinoid signaling | 7 |
| KEGG_PATHWAY | hsa05204 | Chemical carcinogenesis | 6 |
| KEGG_PATHWAY | hsa00061 | Fatty acid biosynthesis | 3 |
| KEGG_PATHWAY | hsa05164 | Influenza A | 9 |
| KEGG_PATHWAY | hsa04911 | Insulin secretion | 6 |
| KEGG_PATHWAY | hsa05222 | Small cell lung cancer | 6 |
| KEGG_PATHWAY | hsa05034 | Alcoholism | 9 |
| KEGG_PATHWAY | hsa04912 | GnRH signaling pathway | 6 |
| KEGG_PATHWAY | hsa05169 | Epstein-Barr virus infection | 7 |
| KEGG_PATHWAY | hsa00360 | Phenylalanine metabolism | 3 |
| KEGG_PATHWAY | hsa04960 | Aldosterone-regulated sodium reabsorption | 4 |
| KEGG_PATHWAY | hsa04110 | Cell cycle | 7 |
| KEGG_PATHWAY | hsa05033 | Nicotine addiction | 4 |
| KEGG_PATHWAY | hsa04916 | Melanogenesis | 6 |
| KEGG_PATHWAY | hsa00220 | Arginine biosynthesis | 3 |
| KEGG_PATHWAY | hsa04961 | Endocrine and other factor-regulated calcium reabsorption | 4 |
| KEGG_PATHWAY | hsa05142 | Chagas disease (American trypanosomiasis) | 6 |
